# Uridine alleviates carbon tetrachloride‐induced liver fibrosis by regulating the activity of liver‐related cells

**DOI:** 10.1111/jcmm.17131

**Published:** 2021-12-30

**Authors:** Wei V. Zheng, Yaqin Li, Xianyi Cheng, Yanwei Xu, Tao Zhou, Dezhi Li, Yu Xiong, Shaobin Wang, Zaizhong Chen

**Affiliations:** ^1^ Intervention and Cell Therapy Center Peking University Shenzhen Hospital Shenzhen China; ^2^ Department of Infectious Disease Peking University Shenzhen Hospital Shenzhen China; ^3^ Department of Minimally Invasion Intervention Peking University Shenzhen Hospital Shenzhen China; ^4^ Health Management Center Peking University Shenzhen Hospital Shenzhen China

**Keywords:** hepatocytes, inflammation, liver fibrosis, uridine, α‐SMA

## Abstract

At present, liver fibrosis is a major challenge of global health. When hepatocyte regeneration cannot compensate for hepatocyte death, it will develop into liver fibrosis in chronic liver disease. Initially, collagen produced by myofibroblasts plays a role in maintaining liver integrity, but excessive collagen accumulation can inhibit the residual liver function, leading to liver failure. At present, many scientists are actively looking for drugs to alleviate liver fibrosis. In the current study, we investigated the potential role of uridine in the treatment of liver fibrosis (uridine is a plant/animal‐derived pyrimidine nucleoside, therefore uridine can also be ingested and absorbed by the body, accompanied by the process of food intake). For this, we systematically studied the effect of uridine on CCl4‐induced liver fibrosis in vitro and in vivo through a series of technologies, such as Western blot, laser confocal scanning microscope, ELISA and immunohistochemistry. The experimental results showed that uridine can effectively reduce the accumulation of collagen in liver. Furthermore, uridine can improve the activity of liver cells and alleviate CCl4‐induced liver injury. Furthermore, uridine can significantly alleviate the risk factors caused by hepatic stellate cell activation, uridine treatment significantly down‐regulated the expression of α‐SMA, collagen type‐I and fibronectin. In conclusion, the current research shows that uridine can alleviate CCl4‐induced liver fibrosis, suggesting that uridine can be used as a potential drug to alleviate liver fibrosis.

## 1 INTRODUCTION

The liver is the largest substantial organ in the human body and the largest digestive organ in the digestive system.^1^ The main function of the liver is to secrete bile, store glycogen and regulate the metabolism of protein, fat and carbohydrates. In addition, the liver also has the functions of metabolism, detoxification and excretion.^1^ In the normal metabolism of the body, the liver is the body's largest digestive gland, so the liver has a strong ability to metabolize foreign substances. At the same time, liver receives its blood supply mainly from the portal vein (approximately 70%) bringing substances absorbed from intestine, therefore it is susceptible to exogenous poisons, alcohol, lipids and drugs. Threats and attacks from viruses, auto‐metabolic diseases and immune‐related diseases cause acute and chronic liver damage (such as inflammation and oxidative stress).^2^ Acute liver injury is generally caused by the liver being stimulated by large doses of chemical poisons, drugs, alcohol and viruses, it can generally be reversed in a short period of time.^3^ However, when the liver is repeatedly stimulated by poisons for a long period of time, chronic liver disease will occur and results in liver fibrosis (even liver), seriously endangering human life and health.^4^

At present, liver disease is still a major factor that endangers human health in the world. Liver damage induced by exogenous poisons, which mainly refer to toxic substances absorbed from the environment, such as carbon tetrachloride (CCl4). In daily life, human body absorbs exogenous toxins through diet, drinking and breathing; these toxins (such as CCl4) enter into the liver via the blood circulation, which in turn leads to acute and chronic liver injury, ultimately leading to hepatic fibrosis.^5^ Therefore, in‐depth study of the pathogenesis of liver fibrosis induced by exogenous toxins and precise therapeutic targets are of practical significance.

Researchers have carried out a series of investigations on the occurrence and development of liver fibrosis. Liver fibrosis is caused by the interaction between persistent liver injury and the accumulation of extracellular matrixes (ECMs). Initially, hepatic stellate cells (HSCs) are activated to differentiate into cells with a myofibroblast‐like phenotype. These myofibroblasts (MFs) secrete collagen and some other matrix to repair liver damage, but excessive accumulation of ECM can cause the repeated liver damage.^6^ In addition, long‐term persistent liver injury inhibits the liver cell function, disrupts the liver balance and results in liver cell dysfunction.^6^, ^7^, ^8^ Now, liver transplantation is the most effective method to treat liver fibrosis. However, due to the difficulty of obtaining transplanted organs, the high cost of surgery and the risk of transplant rejection, liver transplantation cannot be performed in many cases.^9^, ^10^

Many recent studies have reported that bioactive molecules derived from plants or animal can alleviate liver fibrosis. Hibiscus sabdariffa extract showed the protective effects on CCl4‐induced liver fibrosis in rats.^11^ Baicalein administration could inhibit the progress of liver fibrosis.^12^ Furthermore, fucoidan could partly prevent CCl4‐induced liver fibrosis.^13^ In addition, bioactive molecules derived from animal can alleviate liver fibrosis. It has been reported that hepatic damage and fibrosis were reduced in taurine‐supplemented rats.^14^ Furthermore, human serum amyloid P exhibited the protective effect on CCl4‐induced acute liver injury in mice.^15^ But up to now, there is still no relevant drug that can be used clinically.

Uridine, a small and inexpensive pyrimidine nucleoside, is one of the components that constitute the nucleic acid of animal cells.^16^ Uridine is a plant/animal‐derived pyrimidine nucleoside, which is an important material basis for many metabolic processes. Uridine can be ingested and absorbed by the body, accompanied by the dietary process (food intake). A series of studies have shown that uridine has important biological activities.^17^, ^18^ Study has showed that the combined use of uridine and inosine can regulate glucose metabolism in skeletal muscle. It has been reported that uridine injected into knee joints could inhibit arthritis in mice.^17^ Recently, Manish et al reported that uridine treatment ameliorates dextran sulphate sodium‐induced colitis in mice.^18^

In this study, we investigated the effect of uridine treatment on CCl4‐induced liver fibrosis in a mouse model. We revealed that uridine attenuated CCl4‐induced liver fibrosis. In vitro, we further explored the possible mechanism by which uridine relieves the liver fibrosis. It can be found that uridine could alleviate hepatic damage induced by CCL4 in the liver cell model. In addition, in the activated HSC model, we found that uridine can inhibit the expression of α‐SMA and the migration of HSCs. In summary, all these findings highlight the clinical potential of uridine as a treatment strategy for liver fibrosis.

## 2 MATERIALS AND METHODS

### 2.1 Materials

Carbon tetrachloride (CCl4) (#488488) and dimethylsulfoxide (DMSO) (#D2650), Trypsin–EDTA (#T2600000) were obtained from Sigma‐Aldrich. Foetal bovine serum (FBS) (#12483020), phosphate buffered saline (PBS) (#003002) and Dulbecco's modified essential medium (DMEM) (#11885084) were obtained from Thermo Fisher scientific. Anti‐α‐SMA (# ab7817 at 1/500 dilution) and anti‐tumour necrosis factor α (TNF‐α, # 183218 at 1/1000 dilution) were obtained from Abcam. Anti‐tumour growth factor‐β receptor 1 (TGF‐βR1, #ab235178 at 1/600 dilution), anti‐collagen (#ab270993 at 1/800 dilution) and anti‐fibronectin (#ab268020 at 1/400 dilution) were obtained from Abcam Technology (Abcam). Goat anti‐rabbit IgG H&L (Alexa Fluor^®^ 488), goat anti‐rabbit IgG H&L (HRP) (#ab6721 at 1/1000 dilution) and rabbit anti‐mouse IgG H&L (HRP) (#ab6728 at 1/3000 dilution) were obtained from Abcam Technology. Alanine transaminase (ALT), aspartate transaminase (AST), myeloperoxidase (MPO), reduced glutathione/oxidised glutathione assay kit (GSH/GSSG) and superoxide dismutase (SOD) assay kit were from Nanjing Jincheng Institute of Biotechnology. PVDF membrane (# FFP24), 4’6‐diamidino‐2‐phenylindole (DAPI, #C1002) and ECL Plus (#P0018FS) were bought from Beyotime Biotechnology. Enzyme‐linked immunosorbent assay (ELISA) kits for the determination of IL‐1β/TNF‐α/IL‐6/MCP‐1 were purchased from Nanjing Jian‐Cheng Bioengineering Institute. NF‐κB p65 (#8242 at 1/500 dilution), phospho‐NF‐κB p65 (#3033 at 1/600 dilution) and cleavage‐caspase‐3 (#9664 at 1/800 dilution) were purchased from Cell Signalling Technology. All other chemicals used in the current study were of the highest commercial grade (USA).

### 2.2 Experimental animals

All experimental procedures were approved by the institutional and local committee on the care and use of animals of Interventional and Cell Therapy Center, Peking University Shenzhen Hospital (Approval number: 2020‐549), and all animals received humane care according to the National Institutes of Health guidelines. 6‐ to 7‐week‐old male C57BL/6J mice, weighing between 19 ± 2 g, purchased from Beijing Huafukang Laboratory Animal Technology Co., Ltd., laboratory animal production license number: SCXK (Guang‐dong)‐2020‐0006, animals were kept in Peking University Shenzhen Hospital Intervention and Cell Therapy Center (laboratory animal license number: SYXK (Guang‐dong) 2016‐0026). The animal room is kept at room temperature (18°C~22°C), humidity 50 ± 5%, 12/12‐h light/dark cycle. The mice were given free diet, and the experiment was carried out after adaptive breeding for 1 week. All feeding procedures and animal experiment operations were implemented in accordance with the requirements of the Animal Ethics Committee of the Intervention and Cell Therapy Center of Peking University Shenzhen Hospital.

### 2.3 Establishment of mouse liver fibrosis model

The establishment of the experimental mouse model was approved by the Animal Ethics Committee of the Intervention and Cell Therapy Center of Peking University Shenzhen Hospital (Approval number 2020‐549). 30 healthy C57BL/6J mice were chosen (6–7 weeks old, weighing between 19 ± 2 g) and randomly divided them into control group, model group and uridine treatment group. Model group: CCl4 was mixed with olive oil into a 10% oily solvent, the mice from the model group were injected intraperitoneally with the mixed solution at 0.6 μl/g twice per week for 8 weeks; Control group: the C57BL/6J mice were injected intraperitoneally with the same volume of olive oil at the same time point (as negative control); uridine treatment group, the mice were given uridine by drinking water (10–20 mg/kg) for 6 weeks. Twenty‐four hours after the last injection, the eyeballs of experimental animals were removed and blood was collected. The mice were anaesthetized and perfused with saline. The liver tissues were collected for a series of biological analyses as described below.

### 2.4 Cell culture

AML12 and NCTC1469 cells (the normal mouse liver cell line) were cultured in Dulbecco's modified Eagle's medium (DMEM high glucose) with 10% FBS under a humidified atmosphere of 5% CO_2_ at 37°C. HSC‐T6 (rat liver stellate cell line) were obtained from Type Culture Collection of Chinese Academy of Sciences and cultured in DMEM medium with 10% FBS.

### 2.5 Measurement of ROS Level

The cells were grown in six‐well plates and treated with CCl4 (10 mmol/L) for 24 h. After washing, the cells were incubated with 10 μM DCFH‐DA for 45 min. Images from experimental samples were captured by CLSM (Confocal Laser Scanning Microscope, FV3000, Olympus). Furthermore, cells were harvested and analysed by flow cytometer (BD Biosciences). Data were analysed using BD CellQuest software and mean fluorescence intensity was used to represent the ROS levels.

### 2.6 Assay of serum ALT, AST, ALB and TBiL

Blood samples from experimental animals were separated via centrifugation at 2500 *g* for 10 min at 4°C. Automatic biochemical analyser was used to detect the content and concentration of AST, ALT, ALB and TBiL in mouse serum.

### 2.7 Immunofluorescence analysis

Cells (2.5 × 10^4^/well) were grown on cover glass slips in six‐wells plate. Cells were then washed and fixed in 4% paraformaldehyde (PFA) for 20 min. The cells were then treated with 0.1% TritonX‐100 for 10 min and blocked in 5% bovine serum albumin for 60 min. The indicated primary antibodies were then added and incubated for 12 h at 4°C, after which the corresponding second antibodies were added and incubated in the dark for 60 min. DAPI was incubated for another 5 min to stain the nucleus. The cell samples were then observed by CLSM.

### 2.8 Detection of fibrosis‐related biochemical indicators

Blood samples from experimental animals were separated via centrifugation at 3000 rpm for 10 min at 4°C. After the serum samples were collected, the contents of HA and PCIII in the serum of each group mice were analysed by a radioimmunoanalyzer (XH6080).

### 2.9 RT‐PCR

The total RNA was extracted from the cells and tissue using TRIzol (Life Technologies). QRT‐PCR was executed with the QuantiTect SYBR‐Green PCR Kit (Qiagen). Primer sequences used in this study were included in Table S1. The expression of respective genes was normalized to NADPH as an internal control.

### 2.10 H&E staining of mouse liver tissue

The liver tissues from the experimental mice were fixed with 10% formalin solution and soaked for 48 h. Then, the tissue samples were embedded in paraffin and sectioned. Paraffin sections were deparaffinized in xylene. Then, the sections were cooled to room temperature, and the specimens were then immersed into 100%, 100%, 90%, 80% and 70% alcohol for 5 min respectively. The sections were then immersed into the distilled water for 5 min. After rinsing three times with PBS, the sections were immersed into haematoxylin and incubated for 8–10 min. After rinsing sections under running water for 10–20 min, the samples were immersed into eosin to stain for 3 s. The samples were then rinsed under running water for 3–5 min. After the eosin staining, the sections were immersed in xylene to make it transparent for 40 min. After sections became transparent, the sections were sealed with a neutral gum and observed under a microscope.

### 2.11 Masson staining of mouse liver tissue

Liver tissue was fixed with 10% formalin, and then, tissue samples were washed with running water, after which the tissue samples were then routinely dehydrated and embedded. The liver sections were stained with the same amount of iron‐haematoxylin A solution and iron‐haematoxylin B solution according to the manufacturer's instructions and then washed with running water. Tissue samples were then differentiated with alcohol containing 1% hydrochloric acid and continuously rinsed with running water for 5–10 min. Then, the tissues were stained with fuchsin (5–10 min) and observed under a microscope. The tissue samples were treated with molybdophosphoric acid solution and stained with aniline blue. After the slices were treated with acetic acid, the sections were dehydrated rapidly with 95% ethanol and then sealed with neutral gum.

### 2.12 Sirius Red staining

The liver tissue was fixed in 10% formalin, and then, the tissue samples were routinely dehydrated and embedded. The tissue was sliced to the thickness of 6 µm. After dewaxing process, the sections were stained with iron‐haematoxylin staining solution for 10–20 min and then washed with running water for 5–10 min. The sections were stained with Sirius Red staining solution for 1 h and then washed with running water. Tissue sections were routinely dehydrated, and the sections were made transparent by xylene. The sections were then sealed with neutral gum for the microscope observation.

### 2.13 Western Blot analysis

The proteins from liver tissues and cell samples were extracted using the cell lysis buffer with a protease inhibitor cocktail. The protein concentration was determined by BCA Protein Assay Kit (Beyotime Biotechnology). The same amount of extracted protein was subjected to sodium dodecyl sulphate‐polyacrylamide gel electrophoresis (SDS‐PAGE) and transferred to PVDF membrane. The PVDF membrane was blocked with 5% non‐fat milk Tris buffer solution containing 0.05% Tween‐20 (TBS‐T) for 120 min. The membranes were immunoblotted with primary antibody overnight at 4°C, followed by a 1‐h incubation with secondary antibody. Images were recorded and analysed using ImageJ software. β‐actin or GAPDH was used as an internal control.

### 2.14 Cell cycle analysis by flow cytometry

Cell cycle was determined by PI staining via using flow cytometry. Cells were seeded in six‐well plates and cultured in DMEM supplemented with 10% FBS for 24 h, and then were treated with DMSO, CCL4 or CC4+uridine at indicated concentrations for 24 h. Cells were then harvested and fixed, and the cell cycle was then detected by cell cycle analysis kit according to the manufacture's protocol. Percentages of cells within cell cycle compartments (G0/G1, S and G2/M) were determined by flow cytometry (FACS Calibur; Becton, Dickinson and Company). The data were analysed using the software Cell Quest. Results were from triplicate experiments.

### 2.15 Transwell assay

The Matrigel was taken out at −20°C, placed in a −4°C refrigerator overnight and allowed to melt. Matrigel and DMEM was mixed at a ratio of 1:8 (v/v). The diluted Matrigel was then added to the transwell chamber and incubated for 1 h at 37°C. The medium of the HSC was changed to serum‐free DMEM medium and cultured for 3 h. The cells were then resuspended in serum‐free DMEM (1 × 10^6^/ml). The cells were Added in the upper chamber (1 × 10^5^ cells/100 μl). The lower chamber of the blank control group was added with serum‐free DMEM liquid, and the positive control group and the drug treatment group (uridine, 200 μg/ml) were added to the upper chamber with 500 μl DMEM containing 10% FBS. The cells were cultured for 24 h, after which the Matrigel and the cells remaining in the upper chamber were removed by cotton swabs. The cells were washed for three times (5 min/time). The cells were fixed in the methanol solution for 10 min. The cells were then incubated in the haematoxylin dye solution for dyeing for 10 min.

### 2.16 ELISA assay

The cytokine contents in the serum were analysed using an ELISA kit, and the specific experimental process was carried out with reference to the method described in the ELISA kit.

### 2.17 Hepatocyte cell line and apoptosis assay

To avoid the adverse effects of hepatocyte injury produced during perfusion, the mouse hepatocyte cell line (AML12, the mouse hepatocyte cell line) was also used to check the protective effects of uridine on hepatocytes apoptosis caused by CCl4. Before 4 h prior to treatment with 10 mmol/L CCl4 for 10 h, the hepatocytes were treated with uridine (200 µg/ml) or the vehicle (DMSO). Cell apoptosis rate was assessed using a PE Annexin V apoptosis detection kit (#559763, BD) according to the manufacturer's instructions. The cell samples were analysed with a FACSCalibur flow cytometer (Becton‐Dickinson), and cells that are viable are Annexin V‐PE and 7‐AAD negative; cells that are in early apoptosis are Annexin V‐PE positive and 7‐AAD negative; and cells that are in late apoptosis or already dead are both Annexin V‐PE and 7‐AAD positive.

### 2.18 Biochemical analysis

The activities of ALT, AST, SOD or GSH‐Px and the levels of MDA and GSH in serum, liver or cell culture supernatant were determined using commercial kits (Nanjing Jiangcheng Bioengineering Institute) according to the manufacturer's instructions.

In addition, the liver index was calculated as follows: liver weight/body weight ratio.

### 2.19 Statistical analysis

Data are expressed as the mean ± standard deviation (SD). Two‐group comparisons were conducted using the Student's *t* test, and comparisons of the means of 3 or more groups were performed by ANOVA. A value of *p* < 0.05 was considered to indicate a statistically significant difference.

## 3 RESULTS

### 3.1 Uridine alleviated CCL4‐induced liver fibrosis

**FIGURE 1:**
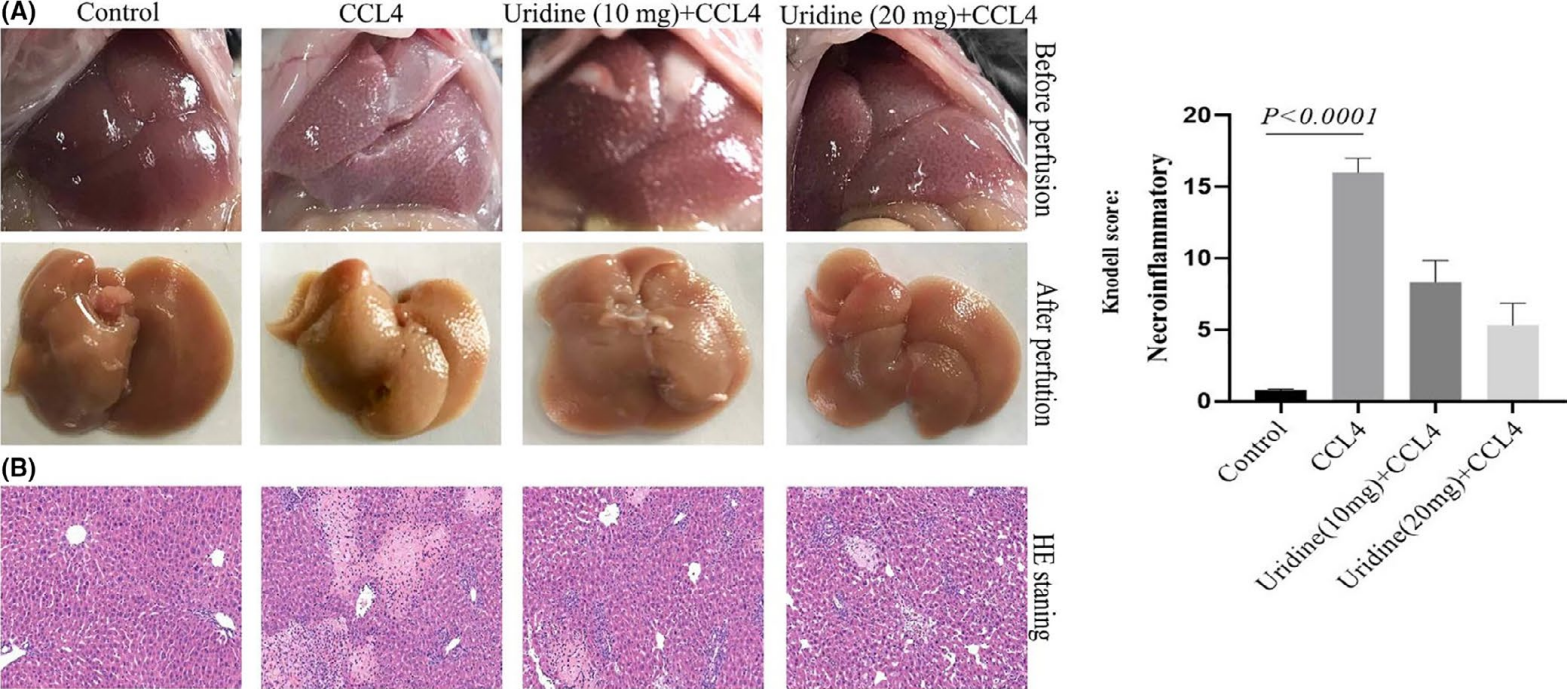
Effect of uridine on the liver morphology change induced by CCL4 treatment. (A) Uridine could attenuate hepatic injury induced by CCl4 in mice. Mice were divided into four group: group 1, vehicle control (no treatment); group 2, model group (CCl4 treatment); group 3, uridine (10 mg/kg) and CCl4‐treated group; group 4, uridine (20 mg/kg) and CCl4‐treated group. (B) Morphology of the liver examined with haematoxylin and eosin (H&E) staining

After mice were treated with CCl4 or oil (control) for 2 weeks, we gave the mice with uridine by drinking water (10 and 20 mg/kg) for 6 weeks. CCl4 or oil treatment was continued for 6 weeks, after which we collected blood and liver tissues from the experimental mice for further analyses. The degree of liver tissue injury was evaluated by liver morphological change and H&E staining. As shown in Figure 1A, it can be observed that the liver surface of CCl4‐treated model group was rough and the cut surface appeared diffuse nodules, and the texture was harder. In the uridine treatment group, the appearance and morphology of the liver tissue were much better compared with the CCl4 model group. In addition, as shown by the H&E staining (Figure 1B), compared with the olive oil administration group, CCL4‐treated group showed necrosis of liver cells, infiltration of inflammatory cells and disordered hepatic cord arrangement. However, liver injury and the degree of inflammation were significantly reduced by the uridine treatment. Additionally, the Knodell score (Knodell histological activity index) also showed that the necroinflammation in the uridine‐treated groups was lower than that of CCL4 group.

### 3.2 Uridine could significantly reduce CCl4‐induced collagen deposition in liver tissue

**FIGURE 2:**
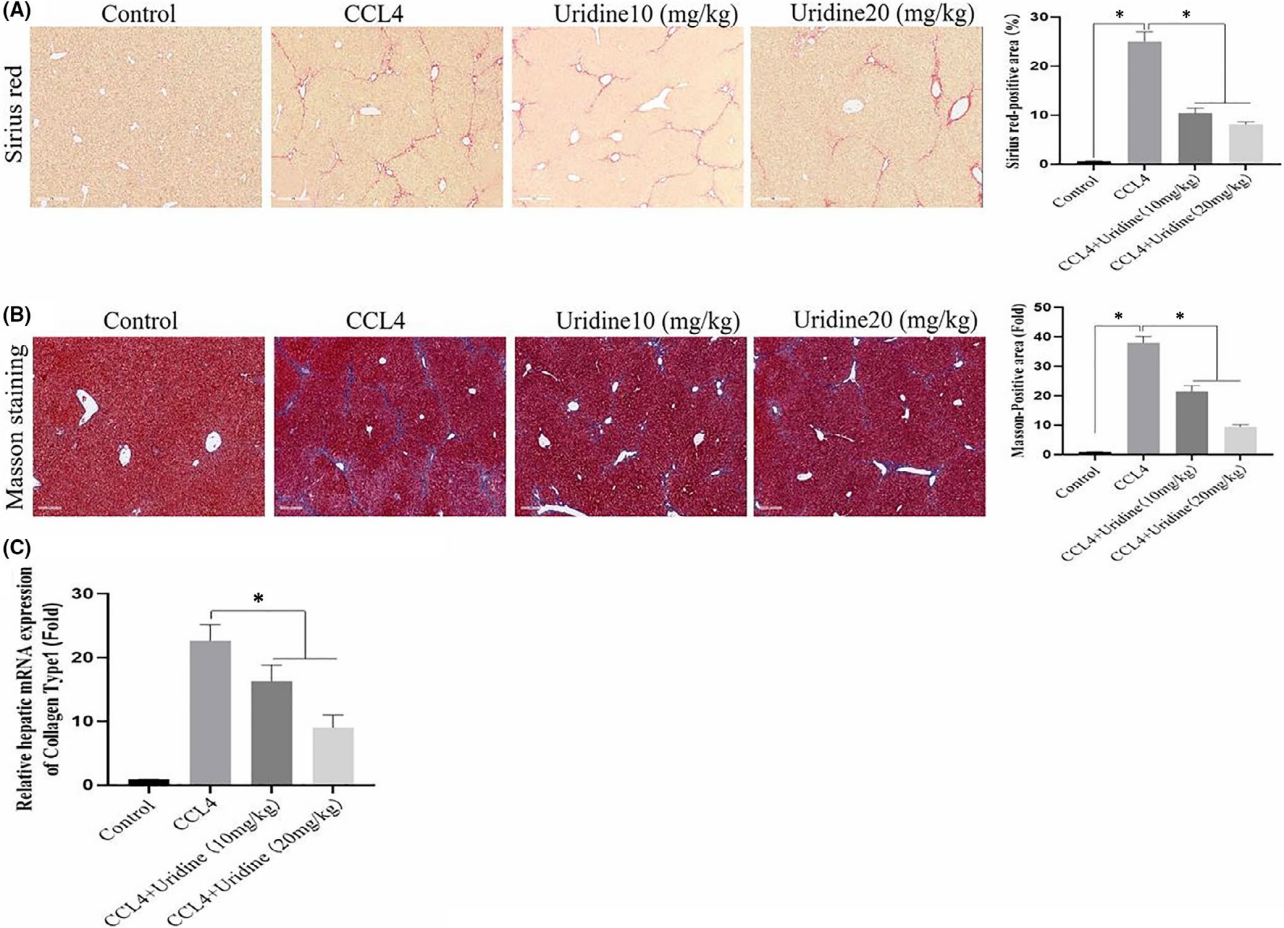
Protective effect of uridine on CCl4‐induced liver damage. (A) Sirius Red staining showed that collagen deposition significantly decreased by uridine treatment. Mice were treated with CCl4, CCl4+uridine or oil as described in materials and methods sections. Data are expressed as the means ± standard deviation. *p < 0.05 (*t*‐test, *n* = 6). (B) Masson staining showed that uridine therapy could attenuate liver fibrosis. Mice were treated with CCl4, CCl4+uridine or oil as described in materials and methods sections. (C) Expression level of the collagen type‐I mRNA was down‐regulated after uridine treatment. Data are expressed as the means ± standard deviation. *p < 0.05 (*t*‐test, *n* = 6). Asterisks indicate significant differences between means (*p* < 0.05)

Sirius Red staining can stain collagen deposited in liver tissue, and collagen is the main component of the ECM. Therefore, Sirius Red staining could be used to determine the degree (extent) of liver fibrosis. For this, we analysed the effect of uridine on collagen deposition by Sirius red staining. As shown in Figure 2A, Sirius red staining showed that the extent of collagen in the CCl4 treatment group was significantly increased compared with the olive oil treatment group. It can be seen that the results of Sirius Red staining showed more fibrosis in CCL4‐treated group (18 ± 2% fibrotic area) compared with control group (0.62 ± 0.12% fibrotic area). However, the uridine treatment significantly alleviated the deposition of collagen (13.4 ± 1.5% fibrotic area for 10 mg/kg uridine treatment, 8.5 ± 1.5% fibrotic area for 20 mg/kg uridine treatment) compared with CCl4‐treated group (18 ± % fibrotic area). At the same time, Masson staining was also performed to evaluate the effect of uridine on liver fibrosis. The staining results confirmed that the collagen deposition in the liver of CCl4‐treated mice was significantly increased, and the collagen deposition increased to ~20‐fold compared with the control group (*p* < 0.05). However, uridine administration alleviated CCl4‐induced collagen deposition, and the collagen deposition decreased to about 11‐fold (10 mg/kg uridine) and 8.6‐fold (20 mg/kg uridine) compared with the control group (Figure 2B).

Type‐I collagen is the main component of collagen in liver fibrosis. We further verified the effect of uridine on type‐I collagen at the RNA level. The type‐I collagen mRNA was increased to about ~23‐fold compared with the control group (*p* < 0.05). However, the uridine treatment group was able to reduce its mRNA to ~16‐fold (10 mg/kg uridine) and ~ninefold (20 mg/kg uridine) when compared to the control group (*p* < 0.05) (Figure 2C). The above results confirm that uridine can reduce the degree of collagen deposition in mice with CCl4‐induced liver fibrosis.

### 3.3 Uridine can significantly reduce the inflammatory response of liver tissue induced by CCl4

**FIGURE 3:**
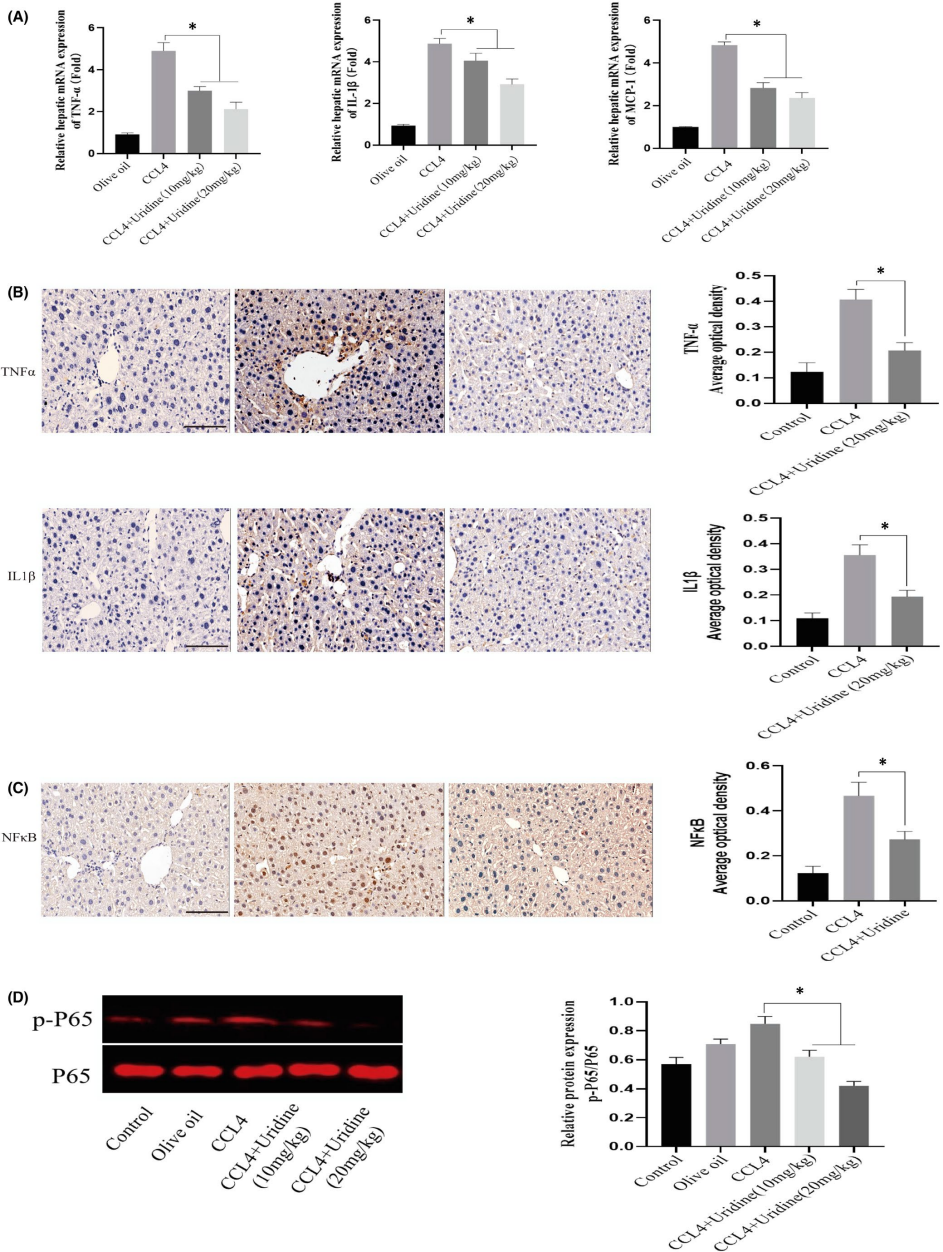
Uridine significantly reduced the inflammatory response caused by CCl4 treatment. (A) The expression level of TNF‐α, IL‐1β and MCP‐1 was down‐regulated by uridine treatment. CCl4 was mixed with olive oil into 10% oily solvent, and injected intraperitoneally into mice at a dose of 2 ml/kg (twice a week for consecutive 8 weeks). Mice were treated with uridine (20 mg/kg) for 6 weeks after treatment with CCl4 for 2 weeks. 24 h after the last injection, the mice were anaesthetized and blood samples were harvested. After perfusion with normal saline, the liver tissues were collected and analysed by RT‐PCR. (B) Inflammatory level was down‐regulated by uridine treatment. Uridine reduced the expression of TNFα and IL‐1β by immunohistochemical analysis. The experimental procedures have been described in detail in the materials and methods section. (C) Inflammatory signalling was down‐regulated by uridine treatment. The expression levels of NF‐κB were analysed by immunohistochemical analysis. (D) The phosphorylation level of P65 was analysed by Western blot analysis. Asterisks indicate significant differences between means (*p* < 0.05)

CCl4 could cause liver damage and inflammation, which is one of the important factors contributing to liver fibrosis.^19^ We therefore evaluated the effect of uridine on inflammation induced by CCl4. As shown in Figure 3A, CCl4 treatment significantly increased the expression of TNF‐α, IL‐1β and MCP‐1 genes in liver tissues by RT‐PCR. We found that compared with the olive oil control group, the TNF‐α, IL‐1β and MCP‐1 gene expression of CCl4‐treated group was significantly increased. However, compared with CCl4‐treated group, the uridine‐treated group significantly down‐regulated the expressions of TNF‐α, IL‐1β and MCP‐1 genes. These results indicated that uridine has an inhibitory effect on the expression of proinflammatory molecules in liver tissue treated by CCl4. In addition, we analysed the effect of uridine on proinflammatory molecules by immunohistochemistry (at the protein level). As indicated in Figure 3B, the expression levels of TNFα and IL‐1β were also reduced by uridine treatment. Additionally, we also studied the effect of uridine on NFκB signalling which is an important signalling pathway responsible regulating proinflammatory molecules expression, and it has been reported that NF‐κB signalling is closely related to liver fibrosis,^27^ and the results showed that CCl4 treatment activated NF‐κB, whereas uridine treatment inhibited the activation of NF‐κB (Figure 3C,D).

### 3.4 Uridine can reduce the level of α‐SMA in mice with liver fibrosis induced by CCl4

**FIGURE 4:**
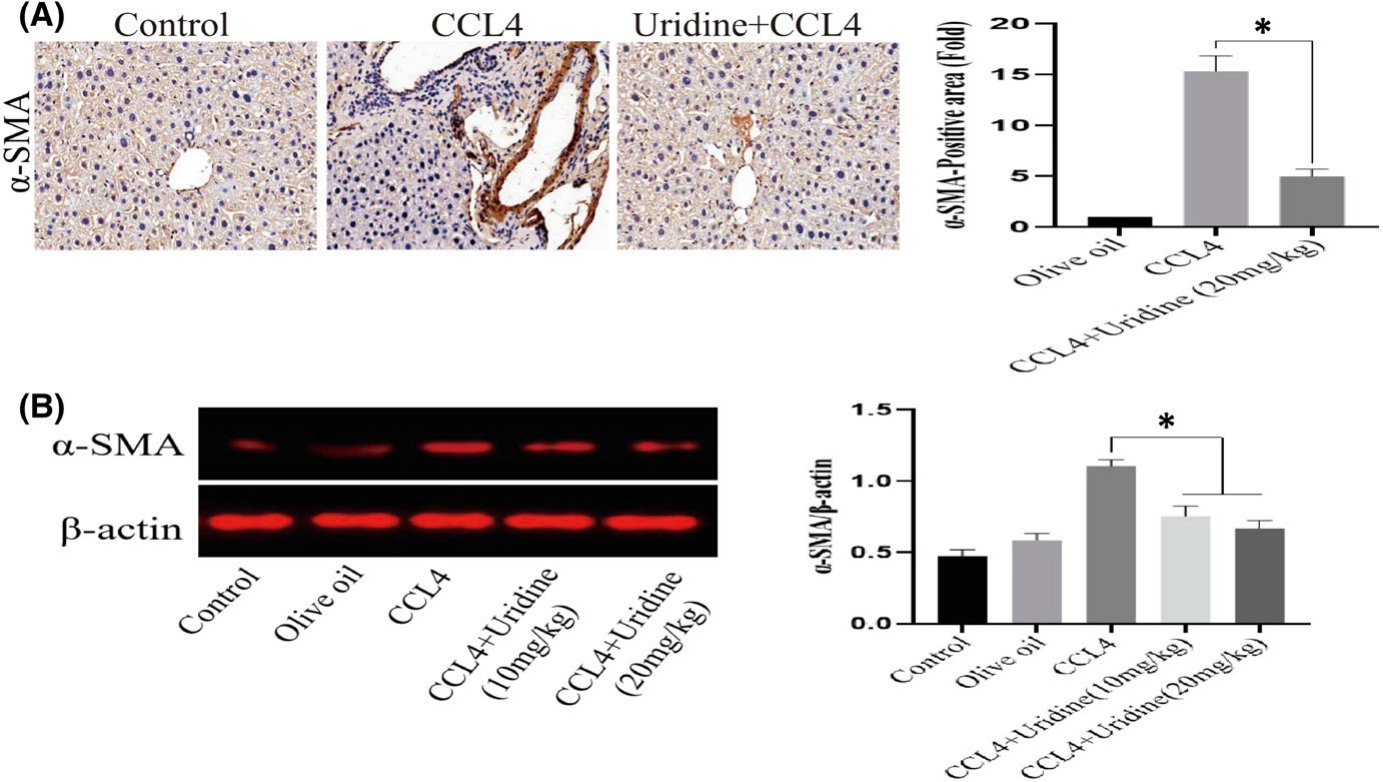
Effect of uridine on the expression of α‐SMA. (A) Uridine treatment inhibited the α‐SMA expression by immunohistochemical staining and indirect immunofluorescence (IFA). CCl4 was mixed with olive oil into 10% oily solvent, and injected intraperitoneally into model group mice at a dose of 0.6 μl/g (twice a week for consecutive 8 weeks). Mice were treated with uridine (20 mg/kg) for 6 weeks after treatment with CCl4 for 2 weeks. 24 h after the last injection, the mice were anaesthetized and liver tissues were harvested and subjective to immunohistochemistry and IFA as described in materials and methods section. (B) The expression level of α‐SMA protein was evaluated by Western blot. The proteins from hepatic tissues were extracted using the tissue lysis buffer. The protein concentration was determined by BCA Protein Assay Kit. The protein samples were subjected to SDS‐PAGE and transferred into PVDF membranes. The blots were imaged and analysed using ImageJ software. Asterisks indicate significant differences between means according to Student's *t*‐test (*p* < 0.05)

HSCs are believed to be the main source of fibrogenic cells. HSC activation is closely related to liver fibrosis. One of the HSC activation markers is α‐SMA. In order to study the effect of uridine on the expression of α‐SMA, as shown in Figure 4A, immunohistochemical staining showed that the α‐SMA was significantly decreased by uridine treatment. The expression of α‐SMA in mouse liver tissue was ~16‐fold higher than that of the control group (*p* < 0.05), whereas the expression of α‐SMA in the uridine treatment group was ~fivefold than that of the control group (*p* < 0.05), indicating uridine can reduce α‐SMA expression in liver tissues of mice with liver fibrosis induced by CCl4 (*p* < 0.05, uridine + CCl4 group vs. CCl4 group). Confocal observation also confirmed this finding. Additionally, this effect of uridine was further verified by the results of Western blot, as shown in Figure 4B, and the relative expression of α‐SMA protein in the liver tissue of the liver fibrosis induced by CCL4 was significantly reduced by uridine therapy.

### 3.5 The effect of uridine on the levels of HA and PIIINP

**FIGURE 5:**
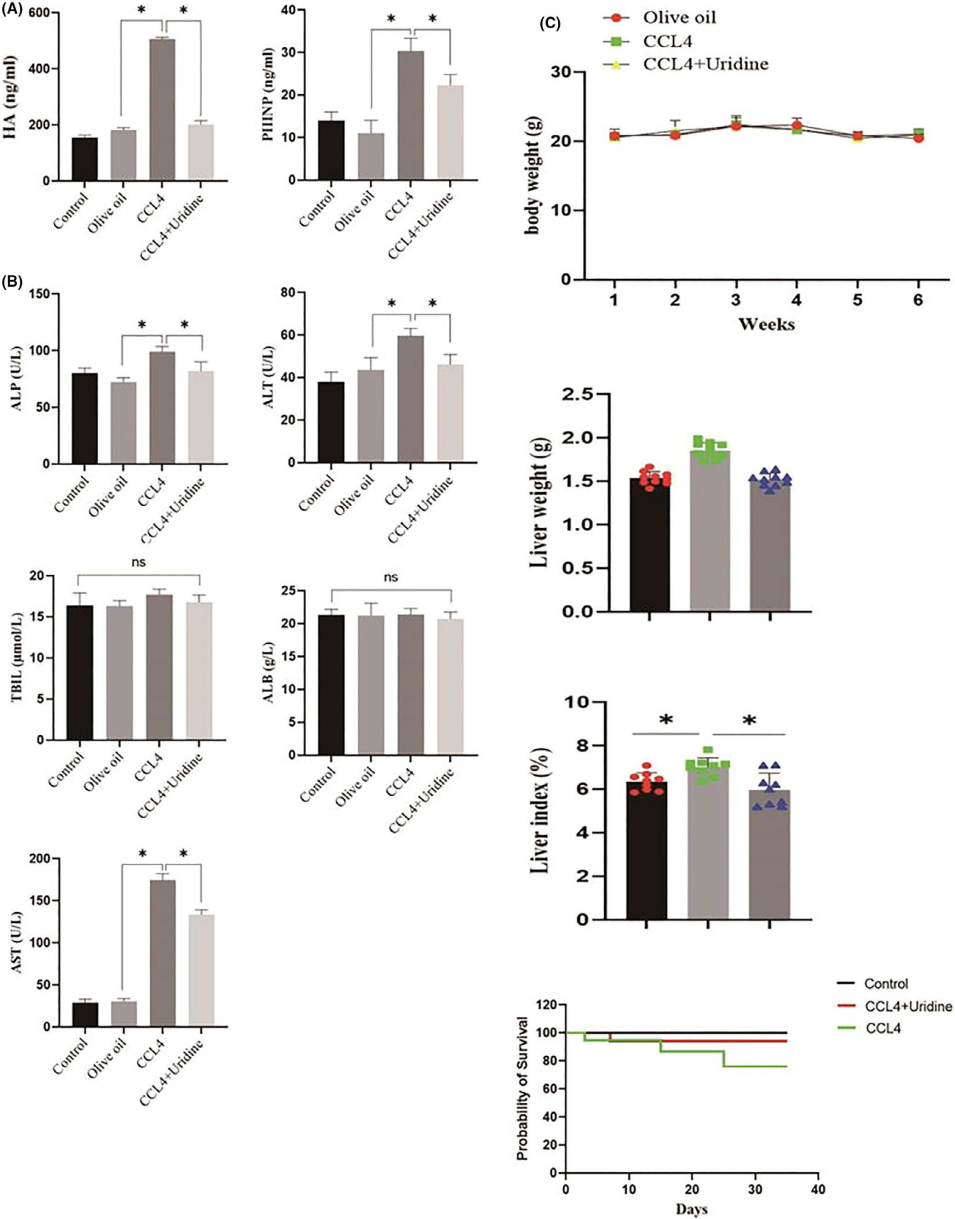
Effect of uridine on the levels of HA and PIIINP in the serum. (A) Uridine treatment reduced the levels of HA and PIIINP in the serum. (B) Effect of uridine on serum ALP, ALT, AST, TBIL and ALB levels in mice. (C) Effect of uridine on body weight, liver weight, liver index and survival curves. Asterisks indicate significant differences between means (*p* < 0.05)

We further analysed the changes in serum levels of fibrosis markers (HA and PIIINP). The serum levels of HA and PIIINP in the CCl4‐treated group were significantly increased (Figure 5A), whereas the levels of HA in the uridine‐treated mice were significantly down‐regulated. Furthermore, there was a significant decrease in serum PIIINP. In addition, we also analysed the changes of liver function and found that uridine could reduce the level of alkaline phosphatase (ALP), alanine aminotransferase (ALT) and aspartate aminotransferase (AST) in mouse serum. In addition, there was no significant difference in serum total bilirubin (TBiL) and albumin (ALB) in all groups (Figure 5B). In addition, body weight, liver weight, liver index and survival curves are shown in (Figure 5C).

### 3.6 Uridine attenuated CCl4‐induced cytotoxicity in the hepatocytes

**FIGURE 6:**
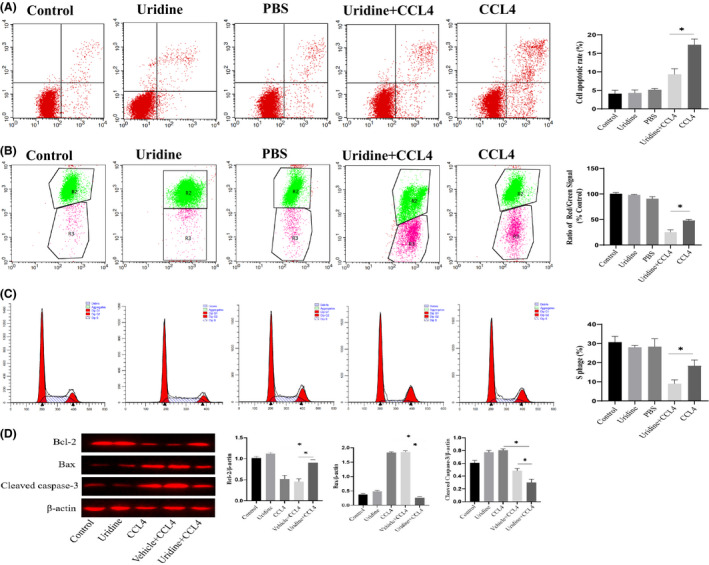
Uridine relieved CCl4‐induced cytotoxicity in the hepatocyte line. (A) uridine inhibited CCl4‐induced apoptosis of liver cells. The apoptosis model was established by 24 h treatment with CCl4 (10 mmol/L). The cells were pre‐treated with uridine (0.2–0.4 mg/mL) for 10 h before CCl4 stimulation. (B) The results from mitochondrial membrane potential analysis indicated that the cell apoptosis rate was down‐regulated by uridine pre‐treatment. (C) Flow cytometry analysis showed that uridine restored the ratio of S phase cells. (D) Effect of uridine on the expression level of Bcl‐2 and Bax and Cleavaged caspase‐3. Asterisks indicate significant differences between means (*p* < 0.05)

The substances released by apoptotic hepatocytes are the important factors that could lead to liver fibrosis.^20^ Therefore, we analysed the effect of uridine on the apoptosis of liver cells induced by CCl4. For this, we used Annexin V‐PE/7‐AAD to measure the apoptotic rates of mouse hepatocyte line (AML12), and the results showed that CCl4 markedly induced hepatocytes apoptosis compared to the control group, and uridine treatment significantly reduced cell apoptosis and exerted a protective effect against CCl4‐induced hepatic cells apoptosis (Figure 6A). Furthermore, the results of mitochondrial membrane potential also showed that uridine could alleviate the apoptosis of CCl4‐induced liver cells (Figure 6B). Flow cytometry analysis showed that uridine restored the ratio of S phase cells (Figure 6C). Additionally, we also measured the expression level of Caspase‐3, Bcl‐2 and Bax, and results shown that the expression of cleavage‐caspase‐3 and Bax was markedly up‐regulated by CCl4 stimulation. In contrast, uridine could significantly reduce the expression of cleavage‐caspase‐3 and Bax expression and up‐regulated Bcl‐2 expression (Figure 6D).

**FIGURE 7:**
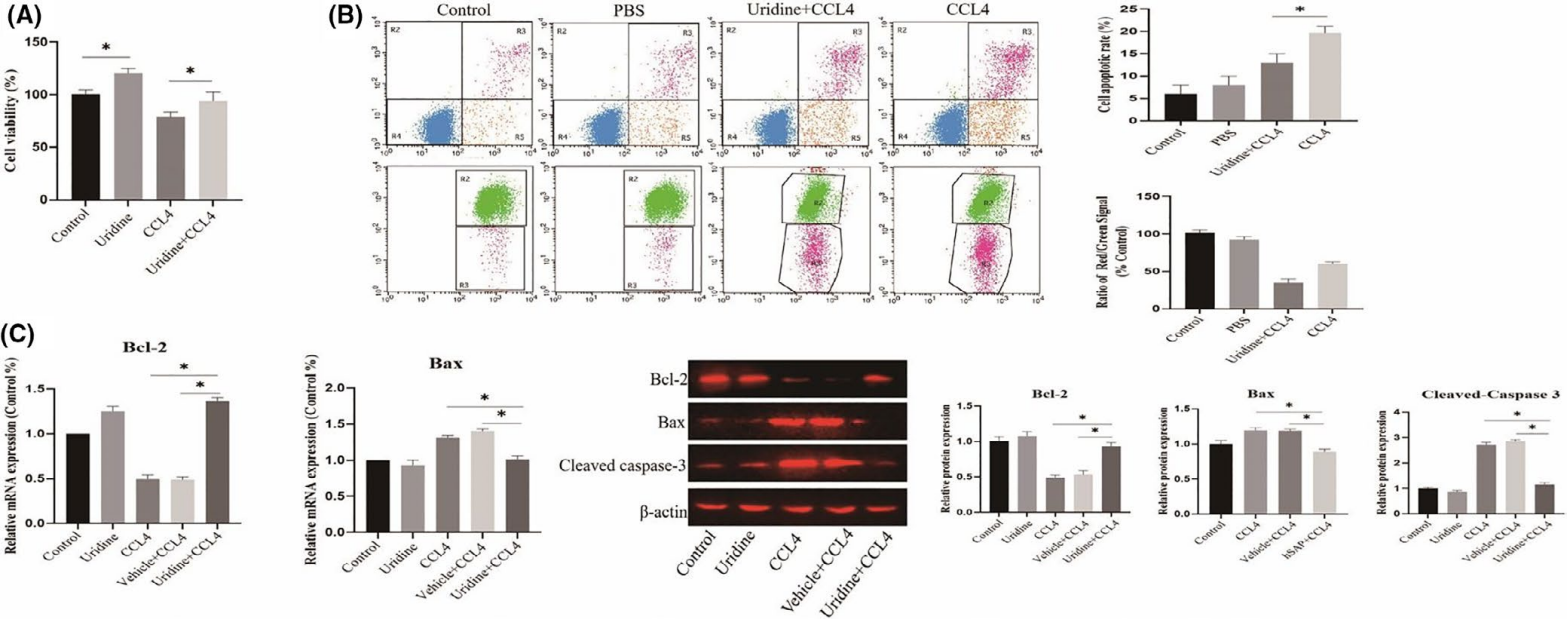
Effect of uridine on the cytotoxicity of CCl4 in freshly isolated hepatocytes. (A) Uridine improves the cell viability of freshly isolated hepatocytes treated with CCl4 (10 mmol/L). (B) Mitochondrial membrane potential analysis showed that the cell apoptosis rate was down‐regulated by uridine treatment. (C) Evaluation of the effect of uridine on the expression level of Bcl‐2 and Bax and Cleavaged caspase‐3. Asterisks indicate significant differences between means (*p* <.05)

In addition to the liver cell line model, we also used freshly isolated hepatocytes as in vitro model to further evaluate the effect of uridine on the cytotoxicity induced by CCl4. For this, MTT assay was firstly performed to evaluate the effect of uridine on the cytotoxicity of CCl4 in freshly isolated hepatocytes. As shown in Figure 7A, cell viability of hepatocytes was reduced when hepatocytes were stimulated with CCl4 (10 mmol/L). However, uridine treatment significantly attenuated the cytotoxic effect induced by CCl4. The cell viability of hepatocytes treated with uridine for 24 h was significantly enhanced compared with the control group. In addition, apoptosis kit and mitochondrial membrane potential analysis also showed that the cell apoptosis rate was down‐regulated by uridine treatment (Figure 7B). Additionally, we also checked the expression of Bcl‐2 and Bax and cleavaged caspase‐3, and results indicated that uridine could alleviate the liver toxicity caused by CCl4 (Figure 7C).

**FIGURE 8:**
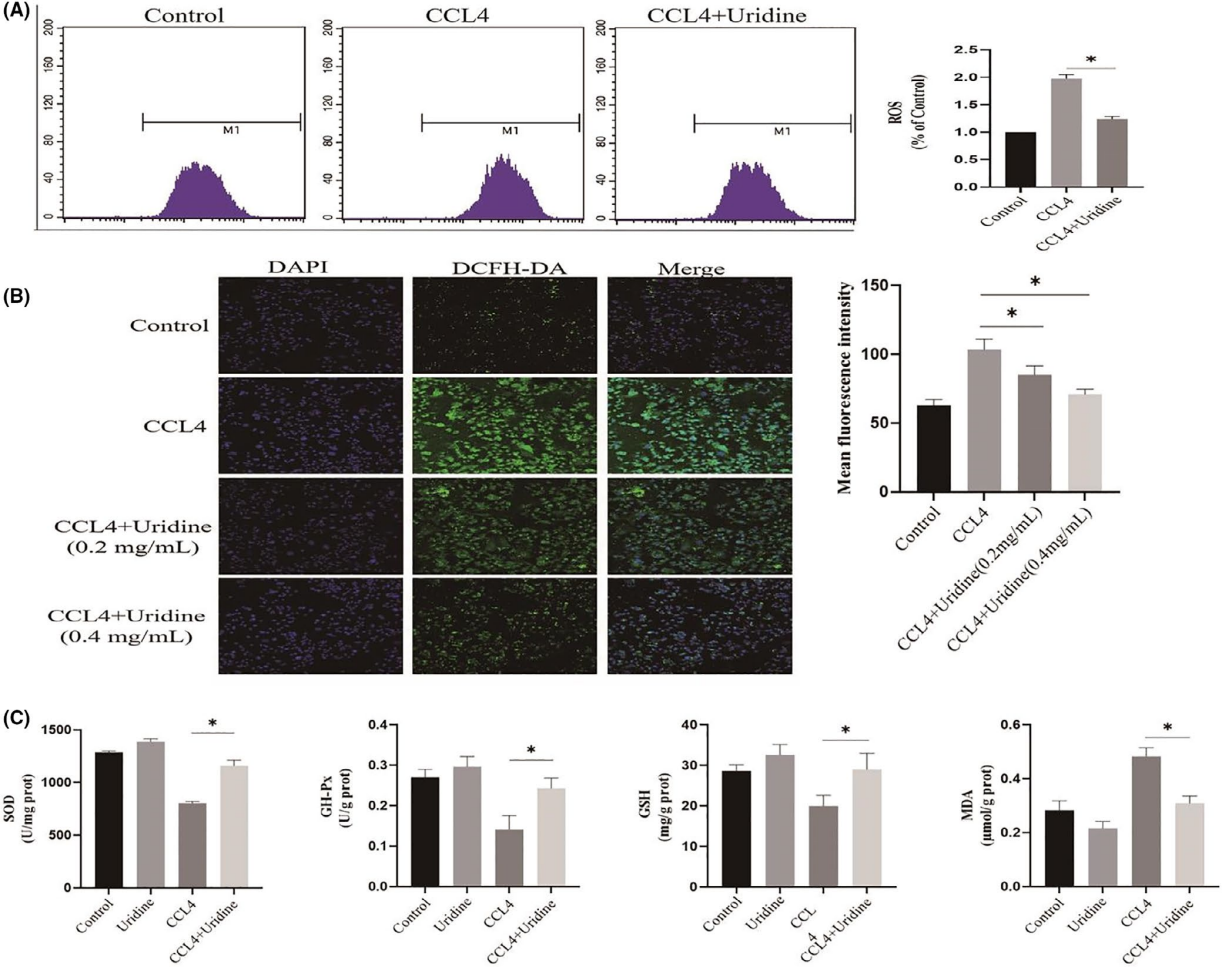
Uridine alleviates oxidative stress caused by CCl4. (A) Uridine pre‐treatment decreased ROS level. (B) Confocal microscopic analysis of ROS level. (C) The effect of uridine on the content of SOD activity, GSH‐Px and GSH. The oxidative stress model was established by 24 h treatment with CCl4 (10 mmoL/L). The cells were pre‐treated with uridine (0.2–0.4 mg/ml) for 10 h before CCL4 stimulation, after which cell samples were collected and subjected to ELISA kit according to manufacturer's instructions. Asterisks indicate significant differences between means (*p* < 0.05)

It has been reported that CCl4‐induced accumulation of free radicals in hepatocytes,^20^ which leads to oxidative stress damage. Therefore, to further investigate the biochemical basis contributing to protective effects of uridine on hepatocytes, we analysed the effect of uridine on CCl4‐induced liver oxidative damage. As shown in Figure 8A, intracellular ROS was detected by flow cytometry, and the results showed that ROS levels were increased to ~twofold compared to the control group. In addition, confocal observation also re‐confirmed this finding (Figure 8B). However, pre‐treatment with uridine reduced intracellular ROS level. Furthermore, SOD activity, GSH‐Px and GSH level in the hepatocytes treated with CCl4 were decreased, and MDA level was increased. In contrast, uridine treatment prevented CCl4‐induced decrease in the SOD, GSH‐Px and GSH level. Furthermore, uridine treatment decreased the MDA level (*p* < 0.05) (Figure 8C).

### 3.7 The effect of uridine on HSC activation and ECM secretion

**FIGURE 9:**
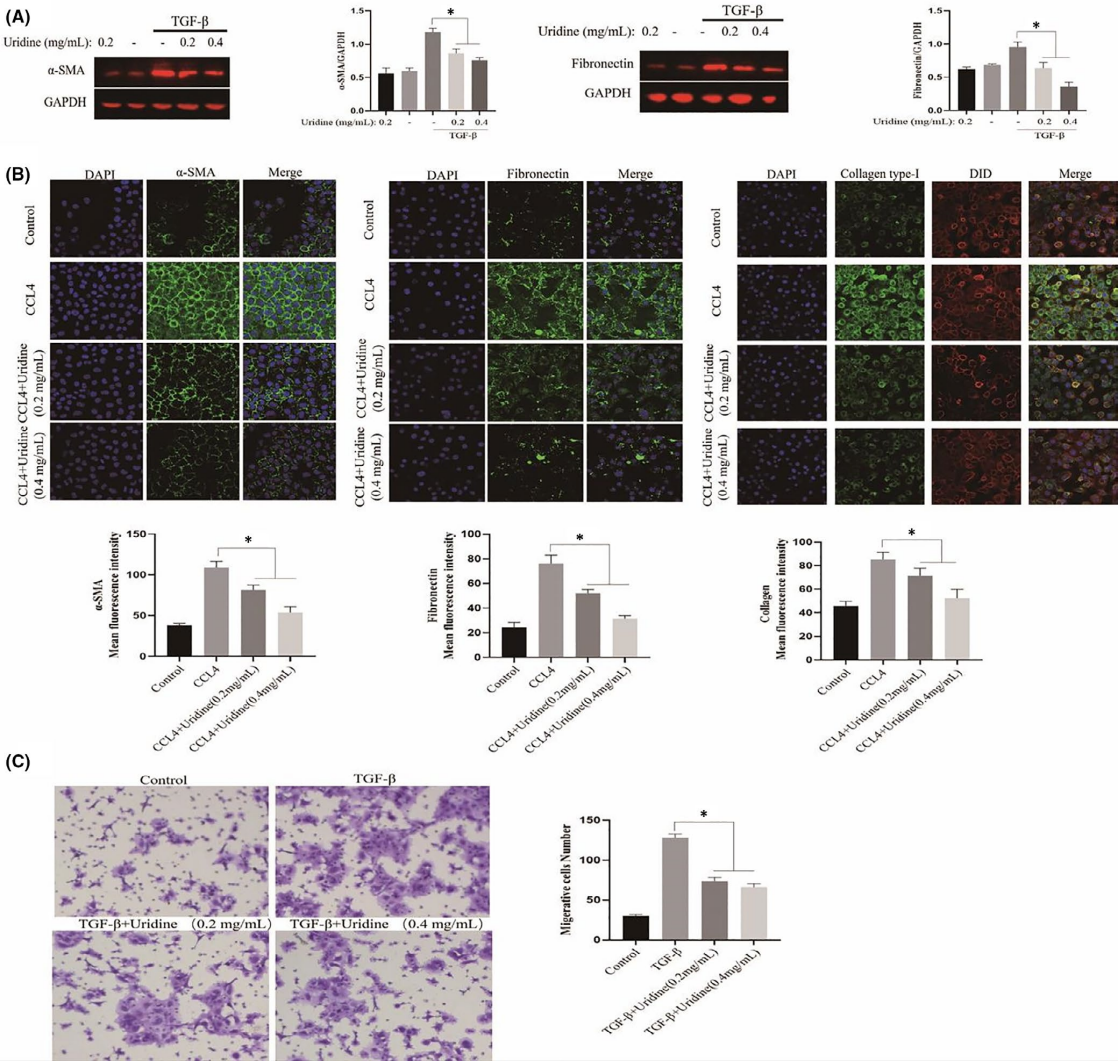
Effect of uridine on activated hepatic stellate cell (HSC). (A) Uridine down‐regulated the expression of α‐SMA in the activated HSCs. An activated cell model of HSC was established by stimulating it with the TGF‐β (25 ng/ml) for 24 h. The cells were pre‐treated with uridine for 10 h before TGF‐β stimulation. (B) Effect of uridine down‐regulated the expression of Collagen type‐I (ColI) and fibronectin (Fn). (C) Uridine inhibited the migration of the activated HSC. Asterisks indicate significant differences between means (*p* < 0.05)

Hepatic stellate cell activation is the main effector cell in the process of liver fibrosis and plays an important role in the occurrence and progression of liver fibrosis. Therefore, we established an activated cell model of HSC by stimulating it with the TGF‐β (25 ng/ml) for 24 h, we then tested the expression of α‐SMA (the marker of HSC activation), and the results showed that TGF‐β increased the protein expression level of α‐SMA in the activated HSC. However, uridine could down‐regulate the expression of α‐SMA, indicating that uridine could inhibit TGF‐β‐induced activation of HSC (Figure 9A). In addition, collagen type‐I (ColI) and fibronectin (Fn) are the important components of the ECM, and excessive ECM deposition (collagen type‐I (ColI) and fibronectin) leads to changes in liver structure, which eventually aggravates liver fibrosis and even develops to liver cirrhosis. It can be seen that uridine could also reduce the expression of ColI and fibronectin in the activated HSC (Figure 9B).

The chemotaxis of activated HSC to chemical substances is enhanced, which is manifested by the movement of HSC under the induction of TGF‐β. In this study, transwell assay was used to detect the effect of uridine on migration/invasion ability of the activated HSC. It can be found that uridine inhibited the migration of the activated HSCs, thereby weakening the chemotaxis of HSCs (Figure 9C).

## 4 DISCUSSION

At present, there is no standard treatment method for liver fibrosis.^30^ The treatment strategy for liver fibrosis mainly includes the following aspects^21^, ^22^: 1) treating the primary disease and removing the continuous damage factors to the liver, for example antiviral treatment for patients with viral hepatitis, to quit drinking for patients with liver disease induced by alcohol, to stop taking liver damage drugs in drug‐induced liver damage patients; 2) to reduce liver inflammation or immune response, to inhibit the activation of HSC induced by damaged or apoptotic liver cells; 3) to reduce scar tissue in the liver formation, to inhibit the deposition of ECM, to inhibit the activation of HSC or promote the apoptosis of activated HSC; 4) to intervene the intracellular or extracellular signalling pathways involved in the process of HSC activation; 5) gene therapy is an alternative method to treat liver fibrosis. In the past ten years, scholars conducting research on gene therapy of liver fibrosis have mainly focussed on genes related to liver metabolism, genes related to liver cell reprogramming and some miRNAs. However, although basic experimental research has made certain achievements in preventing the progress of fibrosis, the effectiveness of most treatment methods has not been confirmed in humans.^2^ This is because clinical trials require liver biopsy to accurately assess changes in liver fibrosis. This process requires long‐term follow‐up studies, and humans may be less sensitive to liver anti‐fibrosis treatment than rodents. The most effective method for the disease is still to treat the disease according to pathogenic factor. Removal of pathogenic factors could effectively restore liver function and liver structure to normal, which in turn reverse the liver fibrosis formation. In addition, the ideal anti‐fibrosis drug should be liver‐specific, has the characteristics of good tolerability under long‐term administration and can effectively reduce excessive collagen deposition without affecting normal ECM synthesis. An important task for the researchers in this field is to explore effective drugs for the treatment of liver fibrosis with fewer side effects. In the current study, we explored and evaluated the effect of the biologically active molecule (uridine) on liver fibrosis, and found that uridine can significantly alleviate liver fibrosis. To the best of our knowledge, this is the first report in this field that uridine can inhibit liver fibrosis.

In the current study, we first constructed a CCl4‐induced liver fibrosis model. Many studies have confirmed that CCl4 can effectively induce liver fibrosis and liver cirrhosis in animal models.^15^, ^26^ This model has been widely adopted by the researchers in this field, because this animal model has a high degree of morphological and functional changes which is similar to human liver fibrosis. In addition, CCl4 is often used to screen potential drugs for anti‐hepatotoxicity.^27^, ^28^, ^29^, ^30^ The mechanism of CCl4‐induced liver injury can be summarized as follows: A) CCl4 toxicity leads to hepatocyte necrosis, mitochondrial damage and aggravate oxidative stress; B) CCl4‐induced proinflammatory (such as TNFα) and pro‐fibrotic cytokines (such as TGF‐β) can further aggravate the degree of liver fibrosis.^27^, ^28^, ^29^ In the current work, our study has shown that uridine can significantly alleviate CCl4‐induced liver fibrosis by a series of experimental results (such as H&E staining, Masson staining and Sirius Red staining). In addition, many recent studies have reported that bioactive molecules derived from plants or animals may alleviate liver fibrosis. Milan et al reported that alpha‐ketoglutarate could relieve liver fibrosis.^23^ Hepatic damage and fibrosis were reduced in taurine‐supplemented rats.^14^ Furthermore, human serum amyloid P exhibited the protective effect of on CCl4‐induced acute liver injury in mice.^15^

Liver fibrosis is a very complex pathological process,^30^ which involves in many different types of cells, such as liver cells, kupffer cells and stellate cells.^11^ We then have to ask the potential molecular mechanism by which uridine relieves liver fibrosis. Liver parenchymal cells are the main cells in the liver, accounting for about 80% of the total number of cells in liver. Hepatic parenchymal cells can secrete inflammatory factors and pro‐fibrosis factors when stimulated by multiple damage factors (such as viruses, drugs and alcohol), they can directly activate HSCs, which is believed as one of the main causes of the liver fibrosis. Therefore, we analysed the effect of uridine on liver cells and found that uridine can alleviate hepatocytes injury caused by CCl4. For example, it can reduce liver cell apoptosis, oxidative stress and inflammatory damage induced by CCl4 treatment.

HSC is the main effector cell in the process of liver fibrosis and plays an important role in the occurrence and progression of liver fibrosis.^24^ Activated HSCs increases the expression levels of fibrosis‐related genes (such as α‐SMA), and significantly enhances ECM secretion, which in turn leads to excessive collagen deposition and finally resulting in liver fibrosis.^24^ Therefore, HSC is also an important therapeutic target in the treatment of liver fibrosis. Therefore, in the current study, we also analysed the effect of uridine on activated HSCs, and the results showed that TGF‐β can increase the α‐SMA expression in HSC. However, uridine treatment can inhibit the α‐SMA expression indicating that uridine may inhibit the HSC activation induced by TGF‐β stimulation. Collagen type‐I (colI) and fibronectin (Fn) are the important components of ECM, and ECM deposition is an important step in the process of liver fibrosis. Excessive ECM deposition leads to changes in the intrahepatic structure, which eventually aggravates liver fibrosis and even develops to liver cirrhosis. In this experiment, we also found that uridine can also reduce the expression of ColI and fibronectin in the activated HSC. These results suggest that the potential mechanism by which uridine inhibits liver fibrosis is complex.

Studies have shown that oxidative stress is also one of the important factors leading to liver fibrosis.^25^ Oxidative stress exhibited the effect on hepatocytes, Kupffer cells (KC) and HSCs during fibrogenesis.^25^ In the current work, we found that uridine showed anti‐oxidative stress effect. These finding suggests that the anti‐oxidative stress pathway may be one of the mechanisms by which uridine could inhibit liver fibrosis.

It has been reported that NF‐κB signalling pathway is closely related to liver fibrosis.^27^ In the current work, we found that uridine treatment could inhibit NF‐κB activation. However, it is interesting that the decreased NF‐κB activity can cause the increased hepatocytes injury and fibrosis.^27^ In contrast, the effect of increased NF‐κB activation on improving liver fibrosis remains unclear. This may be related to the activation degree of NF‐κB signalling. The slight activation of NF‐κB can inhibit liver fibrosis and high activation of NF‐κB can promote the liver fibrosis. Namely, there is probably a threshold beyond which NF‐κB activation improves liver fibrosis (under which NF‐κB activation inhibits liver fibrosis).^27^ In conclusion, we investigated the effect of uridine pre‐treatment on CCl4‐induced liver fibrosis in vitro and in vivo, and we found that uridine attenuated CCl4‐induced hepatic fibrosis. Taken together, the current study indicated that the uridine has the clinical potential in the treatment of liver fibrosis.

## 5 CONCLUSION

In short, we explored the effect of uridine pre‐treatment on CCl4‐induced liver fibrosis in vitro and in vivo experiments, and the results showed that uridine could alleviate CCl4‐induced liver fibrosis, indicating that uridine could be used as a potential drug to alleviate or treat liver fibrosis.

## CONFLICT OF INTEREST

The authors declare no conflict of interest.

## AUTHOR CONTRIBUTIONS

**Wei V. Zheng:** Conceptualization (equal). **Yaqin Li:** Data curation (supporting). **Xianyi Cheng:** Formal analysis (supporting). **Yanwei Xu:** Investigation (supporting). **Tao Zhou:** Methodology (supporting). **Dezhi Li:** Project administration (supporting). **Yu Xiong:** Resources (supporting). **Shaobin Wang:** Software (SUPPORTING). **Zaizhong Chen:** Conceptualization (equal); Data curation (equal); formal analysis (equal).

## Supporting information

Table S1
