# Supplementary material for: Uridine alleviates carbon tetrachloride‐induced liver fibrosis by regulating the activity of liver‐related cells
Source: J Cell Mol Med. 2021 Dec 30;26(3):840–54. doi: 10.1111/jcmm.17131 (PMC8817115; doi:10.1111/jcmm.17131)
Supplement: Supplementary file 1 — Table S1 [file JCMM-26-840-s001.doc]

| **Gene**  **Primer Sequence** |
| --- |
| TNF-α F:TAGCCCACGTCGTAGCAAAC  R:GCAGCCTTGTCCCTTGAAGA [1] |
| Collagen type I  F:CCAGTGGCGGTTATGACTT  R:GCGGATGTTCTCAATCTGC [1] |
| IL-1β [2] F:GGGCCTCAAGGAAAAGAATC  R:TTCTGCTTGAGAGGTGCTGA |
| MCP-1 F:GCTCTCAGGATCCTATGCAGGTCCCTGTC  R:TGATGGGATCCGTCCGAGTCACACTA |
| GAPDH F:ACCCAGAAGACTGTGGATGG  R: CACATTGGGGGTAGGAACAC |
| α-SMA F:CTGACAGAGGCACCACTGAA  R:GAAGGAATAGCCACGCTCAG |
| BCL-2 F:GACAGAAGATCATGCCGTCC  R:GGTACCAATGGCACTTCAAG |
| Bax F:GAGCTGACCTTGGAGC  R: GACTC-CAGCCACAAAGATG [3] |

**Supplementary table 1. List of primer sequence**

[1] Shi, H., Lo, T. H., Ma, D., Condor, B., Lesmana, B., Parungao, R. J., ... & Wang, Y. (2020). Dihydrotestosterone (DHT) Enhances Wound Healing of Major Burn Injury by Accelerating Resolution of Inflammation in Mice. International journal of molecular sciences, 21(17), 6231.

[2] Zhang, M., Qi, Y., Li, H., Cui, J., Dai, L., Frank, J. A., ... & Chen, G. (2016). AIM2 inflammasome mediates Arsenic-induced secretion of IL-1 β and IL-18. Oncoimmunology, 5(6), e1160182.

[3] Vukosavic, S., Dubois‐Dauphin, M., Romero, N., & Przedborski, S. (1999). Bax and Bcl‐2 interaction in a transgenic mouse model of familial amyotrophic lateral sclerosis. Journal of neurochemistry, 73(6), 2460-2468.
